# Methylene Blue Reduced Abnormal Tau Accumulation in P301L Tau Transgenic Mice

**DOI:** 10.1371/journal.pone.0052389

**Published:** 2012-12-20

**Authors:** Masato Hosokawa, Tetsuaki Arai, Masami Masuda-Suzukake, Takashi Nonaka, Makiko Yamashita, Haruhiko Akiyama, Masato Hasegawa

**Affiliations:** 1 Department of Dementia and Higher Brain Function, Tokyo Metropolitan Institute of Medical Science, Tokyo, Japan; 2 Department of Psychiatry, Graduate School of Comprehensive Human Sciences, University of Tsukuba, Tsukuba, Japan; 3 Department of Pathology and Cell Biology, Tokyo Metropolitan Institute of Medical Science, Tokyo, Japan; McGill University, Canada

## Abstract

In neurodegenerative disorders, abnormally hyperphosphorylated and aggregated tau accumulates intracellularly, a mechanism which is thought to induce neuronal cell death. Methylene blue, a type of phenothiazine, has been reported to inhibit tau aggregation in vitro. However, the effect of methylene blue in vivo has remained unknown. Therefore, we examined whether methylene blue suppresses abnormal tau accumulation using P301L tau transgenic mice. At 8 to 11 months of age, these mice were orally administered methylene blue for 5 months. Subsequent results of Western blotting analysis revealed that this agent reduced detergent-insoluble phospho-tau. Methylene blue may have potential as a drug candidate for the treatment of tauopathy.

## Introduction

In neurodegenerative disorders such as Alzheimer’s disease, corticobasal degeneration, and supranuclear palsy, the microtubule–associated protein tau is abnormally phosphorylated and redistributed into paired helical filaments (PHFs) forming neurofibrillary tangles, a process that correlates with pyramidal cell destruction and dementia. Abnormal tau accumulation is characterized by hyperphosphorylation, conformational change and aggregation with changes in solubility.

Wischik et al. have identified a nonneuroleptic phenothiazine which reverses the proteolytic stability of protease-resistant PHFs by blocking tau-tau binding through the repeat domain [1]. Moreover, phenothiazines, including methylthioninium chloride (methylene blue (MB)), polyphenols and porphyrins, inhibited heparin-induced tau filament formation in vitro [2]. Based on these results, tau aggregation inhibitors are considered to be strong candidates for the treatment of tauopathy.

TauRx Pharmaceuticals recently announced the completion of MB phase II clinical trials. They conducted MB dosing and efficacy studies involving 321 people with mild to moderate Alzheimer’s disease. Over a 50-week period, the cognitive decline of those on the drug appeared to be 81% slower than those taking a placebo. These results were presented at a conference [3] but have not been published. Currently, a large-scale phase III trial is in planning [4].

To date, there are two reports on the effect of MB on tau aggregation in vivo. In one study, MB did not alter abnormal tau phosphorylation and failed to inhibit tau-dependent neuronal cell toxicity in zebrafish [5]. The other study employed a distinct tau transgenic mouse line and mice received a 2–3-week treatment with oral MB. In this latter study, MB acted as a tau aggregation inhibitor. Although, these findings have been described in brief in a conference abstract, the details are unavailable [6].

More recently, Congdon et al. demonstrated that MB could induce autophagy in primary neurons, organotypic slice cultures and tau transgenic mice (JNPL3) [7]. They also showed a 2 week oral administration of MB attenuated the total tau levels in the absence of significant changes in sarkosyl-insoluble tau levels.

In the present study, we investigated whether MB could reduce abnormal tau accumulation by carrying out long-term oral administration of MB using tau transgenic mice with the P301L mutation as a tauopathy model. Our results suggested that oral intake of MB could be a potential treatment for tauopathy.

## Materials and Methods

### Ethics Statement

This study was carried out in strict accordance with the recommendations provided in the Guide for the Care and Use of Laboratory Animals of the Ministry of Health, Labour and Welfare of Japan and the Ministry of Education, Culture, Sports, Science and Technology of Japan. The protocol was approved by the Committee on the Ethics of Animal Experiments of the Tokyo Metropolitan Institute of Medical Science (Permit Numbers: 22–23 and 11-028). All experiments were performed under sodium pentobarbital anesthesia and every effort was made to minimize suffering.

### Animals

P301L tau transgenic mice (JNPL3) [8] were purchased from Taconic (USA) via IBL (Japan). The experiments utilized 44 female hemizygote tau mice. The mice were reared in the animal facility of Tokyo Metropolitan Institute of Medical Science under standard conditions at 24±2°C and were maintained on a commercial diet supplied ad libitum.

The transgenic mice [8] aged 8–11 months were divided into 3 groups: the first group (14 mice) was given water alone, the second group (15 mice) was given water containing 2 µg/ml MB and the third group (15 mice) was given water containing 6 µg/ml MB. All were maintained on their respective regimens for 5 months. The daily MB intake was estimated to be about 0.3 or 1 mg/kg/day per mouse, on the assumption that a mouse weighs 30 g and takes in 5 ml of water a day.

At the end of the experimental period, mice were sacrificed under quick anesthesia with 200 mg/kg body weight of sodium pentobarbital delivered intraperitoneally and their brains were quickly removed. Brains of each group were cut along the sagittal plane and the left hemisphere was frozen and stored at −80°C for biochemical analyses. The right hemisphere was fixed in 4% paraformaldehyde in 0.1 M phosphate buffer for 48 hours in the cold. Brain blocks were then transferred to a maintenance solution of 15% sucrose in 0.01 M phosphate-buffered saline (PBS), pH 7.4.

### Analysis of Tau Deposition

Deposition of tau was analyzed using immunohistochemical staining with AT8 antibody (recognizes phosphorylation at both serine 202 and threonine 205) and MC-1 antibody. Biotinylation of MC-1 antibody was performed using a Zenon Mouse IgG Labeling Kit (Molecular Probes, Inc. Eugene, OR, USA) according to the manufacturer’s instructions. For immunohistochemistry, sagittal sections were cut serially on a freezing microtome at 30 µm thickness, collected in the maintenance solution, and immunostained as free-floating sections. Following a pretreatment with 0.5% H_2_0_2_ for 30 min to eliminate endogenous peroxidase activity, sections were incubated for 72 hours with AT8 antibody (Biotinylated-AT8, 1∶1,000, Innogenetics, Ghent, Belgium) or overnight with biotinylated MC-1 antibody (1∶100, a generous gift of Dr. Davies) [9] diluted in PBS containing 0.3% Triton X-100 (PBS-Tx). The antibody labeling was visualized by incubation with avidin-biotinylated horseradish peroxidase (HRP) complex (ABC Elite, 1∶1,000, Vector Laboratories, Burlingame, CA, USA) for 3 hours, followed by incubation with a solution containing 0.01% 3,3′-diaminobenzidine (DAB), 1% nickel ammonium sulfate, 0.05 M imidazole and 0.00015% H_2_0_2_ in 0.05 M Tris-HCl buffer, pH 7.6. Counter nuclear staining was performed with Kernechtrot stain solution (Merck, Darmstadt, Germany). The sections were then rinsed with distilled water, mounted on glass slides, treated with Xylene, and coverslipped with Entellan (Merck). Photographs were taken with an Olympus VS120-S5 (Olympus, Tokyo, Japan) or a BX51 microscope (Olympus).

### Sequential Fractionation of Brain Extracts

Frozen left hemispheres (approximately, 0.2 g) were homogenized in 10 volumes of buffer H (10 mM Tris-HCl, pH 7.5, 0.8 M NaCl, 1 mM ethylene glycol bis-N, N, N’, N’-tetraacetic acid, 1 mM dithiothreitol). The hemisphere included the olfactory bulb, cerebral cortex, striatum, thalamus, hypothalamus, cerebellum, midbrain, pons, medulla oblongata and the upper part of the spinal cord. The method used for sequential fractionation of brain extracts was originally described by Greenberg et al. [10]. Briefly, the brain homogenate was centrifuged at 100,000×g for 20 min at 4°C, and the supernatants were collected as the Tris-soluble fraction. The resultant pellet was homogenized in 10 volumes of buffer H, followed by an incubation for 30 min at 37°C with 1% Triton X-100. The homogenate was then centrifuged at 100,000×g for 20 min at 4°C. The Triton X-100 insoluble pellet was sonicated in 5 volumes of buffer H containing 1% sarkosyl and centrifuged at 100,000×g for 20 min at 4°C. The pellet was then sonicated in 1 volume of SDS-PAGE sample buffer.

### Immunoblotting Analysis

For immunoblotting, brain extracts from the tau mice were boiled for 5 minutes with SDS-PAGE sample buffer (60 mM Tris-HCl, pH 6.8, containing 2% SDS, 10% glycerol, 0.025% bromophenol blue and 5% mercaptoethanol) and loaded onto a 10% acrylamide minigel. Loaded samples were electrophoresed for 45 minutes at 200 V with molecular weight markers (Bio-Rad, Hercules, CA, USA). Electrophoresed proteins were transferred onto a polyvinylidene difluoride membrane (Millipore, Billerica, MA, USA) for 60 minutes at 200 mA. The printed membranes were blocked with 3% gelatin for 1 hour and then incubated in a primary antibody solution (HT7, 1∶3,000, Innogenetics, AT8, 1∶3,000, Innogenetics, or anti-α-tubulin, 1∶10,000, Sigma, St. Louis, MO, USA) overnight at room temperature. Following incubation with a secondary antibody (1∶20,000, Bio-Rad), immunoreactivity was detected by the chemiluminescence method using an ECL plus Western Blotting Detection Kit (GE Healthcare UK Ltd., Buckinghamshire, England) or SuperSignal West Dura Extended Duration Substrate (Pierce, Rockford IL, USA) and was visualized with LAS-3000 (Fujifilm, Tokyo, Japan). For quantitative measure of band intensity, α-tubulin was used as an internal control for protein concentration.

### Statistical Analyses

The data are presented as medians (interquartile range) [range]. The significance of difference between values was estimated by means of Kruskal-Wallis analysis of variances with post hoc Steel’s method. P<0.05 was considered to indicate a statistically significant difference.

## Results

### Immunoblotting Analysis

**Figure 1:**
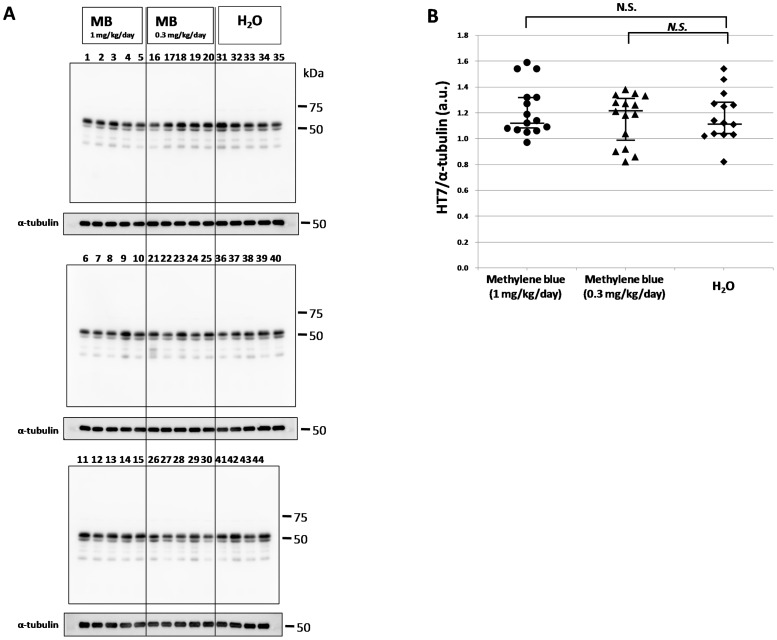
Immunoblotting analysis of total tau in the Tris-soluble fraction. (A) Immunoblotting analysis was visualized using HT7 antibody for the Tris-soluble fraction. The numbers indicate individual mice: 1–15, MB 1 mg/kg/day group; 16–30, MB 0.3 mg/kg/day group; and 31–44, water only group. Molecular weight markers are shown on the right (kDa). For quantitative measure of band intensity, α-tubulin was used as an internal control for protein concentration. (B) A comparison of the relative total tau (HT7) expression levels of the MB-treated groups and the water only group. The data were compared with the HT7 band intensity, which was normalized with α-tubulin. The central lines indicate medians and the vertical lines represent 25^th^ and 75^th^ percentiles. a.u., arbitrary unit. N.S., no significant difference.

**Figure 2:**
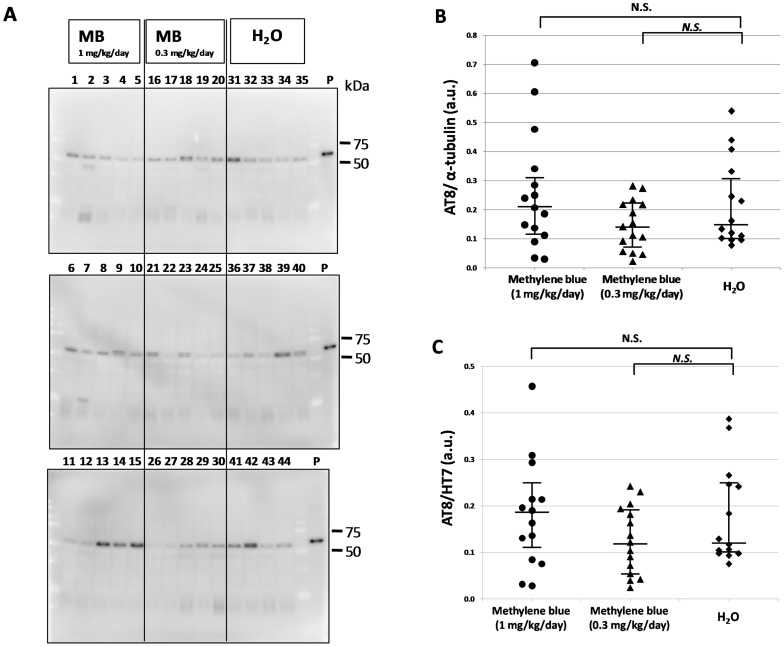
Immunoblotting analysis of phosphorylated tau in the Tris-soluble fraction. (A) Immunoblot analysis was visualized using AT8 antibody for the Tris-soluble fraction. The numbers indicate individual mice: 1–15, MB 1 mg/kg/day group; 16–30, MB 0.3 mg/kg/day group; and 31–44, water only group. Molecular weight markers are shown on the right (kDa). P, positive control (P301L tau transgenic mouse, 20 month-old female). (B) A comparison of relative phosphorylated tau (AT8) expression levels of the MB-treated groups and the water only group. The data were compared with the AT8 band intensity, which was normalized with α-tubulin. (C) A comparison of the relative phosphorylated tau (AT8)/total tau (HT7) levels of the MB-treated groups and the water only group. The data were compared with the AT8 band intensity, which was normalized with the total tau (HT7) band intensity. The central lines indicate medians and the vertical lines represent 25^th^ and 75^th^ percentiles. a.u., arbitrary unit. N.S., no significant difference.

**Figure 3:**
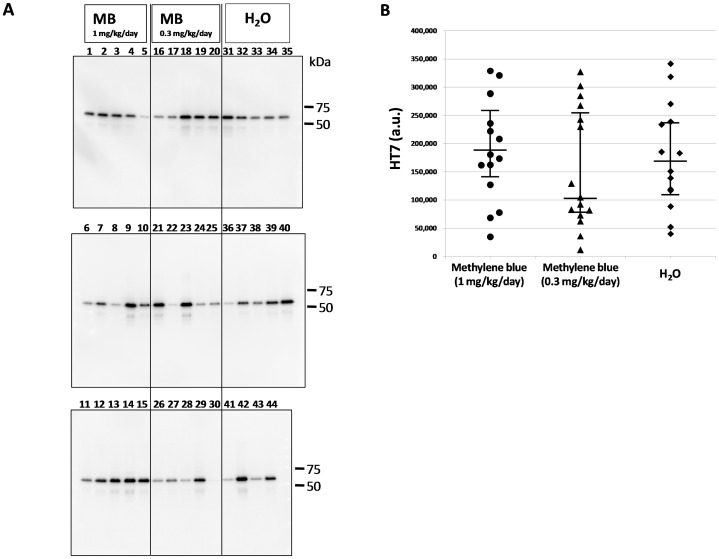
Immunoblotting analysis of total tau in the sarkosyl-insoluble fraction. (A) Immunoblot analysis was visualized using HT7 antibody for the sarkosyl-insoluble fraction. The numbers indicate individual mice: 1–15, MB 1 mg/kg/day group; 16–30, MB 0.3 mg/kg/day group; 31–44, water only group. Molecular weight markers are shown on the right (kDa). (B) A comparison of relative total tau (HT7) expression levels in the sarkosyl-insoluble fraction of the MB-treated groups and the water only group. The data were compared with the HT7 band intensity. The central lines indicate medians and the vertical lines represent 25^th^ and 75^th^ percentiles. a.u., arbitrary unit.

**Figure 4:**
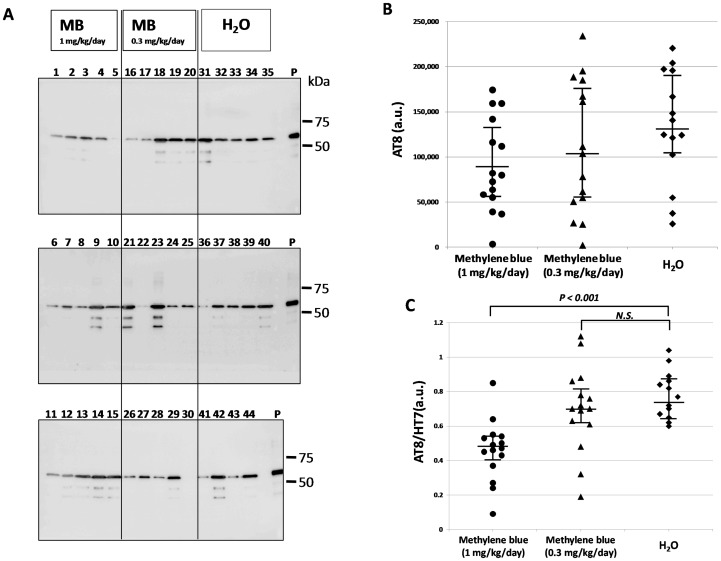
Immunoblotting analysis of abnormal tau in the sarkosyl-insoluble fraction. (A) Immunoblotting analysis was visualized using AT8 antibody for the sarkosyl-insoluble fraction. The numbers indicate individual mice: 1–15, MB 1 mg/kg/day group; 16–30, MB 0.3 mg/kg/day group; and 31–44, water only group. Molecular weight markers are shown on the right (kDa). P, positive control (P301L tau transgenic mouse, 20 month-old female). (B) A comparison of relative AT8 expression levels of the MB-treated groups and the water only group. The data were compared with the AT8 band intensity. (C) A comparison of relative phosphorylated tau (AT8)/total tau (HT7) levels of the MB-treated groups and the water only group. The data were compared with the AT8 band intensity, which was normalized with the total tau (HT7) band intensity. The central lines indicate medians and the vertical lines represent 25^th^ and 75^th^ percentiles. P<0.01 was considered to represent a statistically significant difference. a.u., arbitrary unit. N.S., no significant difference.

To investigate the effect of methylene bule on tau accumulation, P301L tau transgenic mice were administered MB for 5 months starting at 8–11 months of age. Brains were then collected and sequential protein extraction and immunoblotting were performed (Figs. 1, 2, 3, 4). Total tau in the Tris-soluble fraction was detected by HT7 antibody (Fig. 1A), and there were no prominent differences among the three groups by quantitative analysis of band intensities, which was normalized with α-tubulin (Fig. 1B). Phosphorylated tau in the Tris-soluble fraction was also detected by AT8 antibody (Fig. 2A). The data were compared with the AT8 band intensity, which was normalized with α-tubulin (Fig. 2B) or with the total tau (HT7) band intensity (Fig. 2C). There were also no significant differences in phosphorylated tau in the Tris-soluble fraction among the three groups (Figs. 2B and 2C).

Phosphorylated tau and total tau in the sarkosyl-insoluble fraction were visualized again by Western blotting using HT7 antibody (Fig. 3A) and AT8 (Fig. 4A), respectively. The HT7 (Fig. 3B) and AT8 (Fig. 4B) band intensities were measured. Then, we compared the AT8 band intensity normalized by the total tau (HT7) band intensity and were expressed as medians (interquartile range) [range]. The AT8/HT7 ratio was 0.47 (0.40–0.53) [0.09–0.85] (n = 15) in the MB group given 1 mg/kg/day, 0.70 (0.62–0.82) [0.19–1.12] (n = 15) in the MB group given 0.3 mg/kg/day, and 0.74 (0.65–0.86) [0.60–1.04] (n = 14) in the group given water alone (Fig. 4C). The Kruskal-Wallis test followed by Steel’s multiple comparison test was used for statistical analyses, and there was a significant difference between the group given MB 1 mg/kg/day and the group given water alone (P<0.001). There was no significant difference between the 0.3 mg/kg/day group and the water only group (P  = 0.422). Immunoblotting analysis revealed that the level of abnormally phosphorylated tau accumulation was decreased in the P301L tau mice given MB as compared with the P301L tau mice given water alone.

### Analysis of Tau Deposition

**Figure 5:**
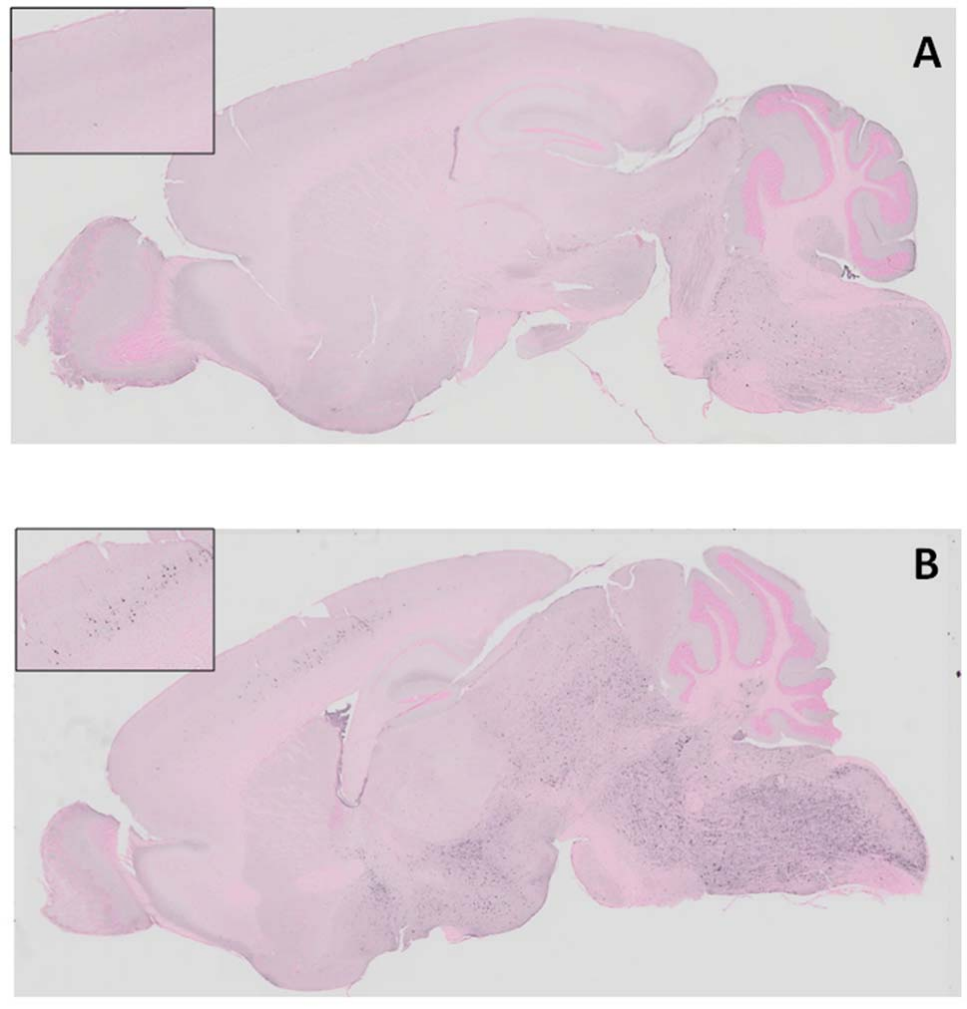
Immunohistochemical staining of abnormal tau. (A) An AT8 immunoreaction was observed only in spinal cord, medulla oblongata and pons of a mouse with a low AT8/HT7 ratio. (B) AT8-positive cells were seen in spinal cord, medulla oblongata, pons, midbrain, hypothalamus and cerebral cortex of a mouse with a high AT8/HT7 ratio. Each insert shows the cerebral cortex of the mouse brain.

**Figure 6:**
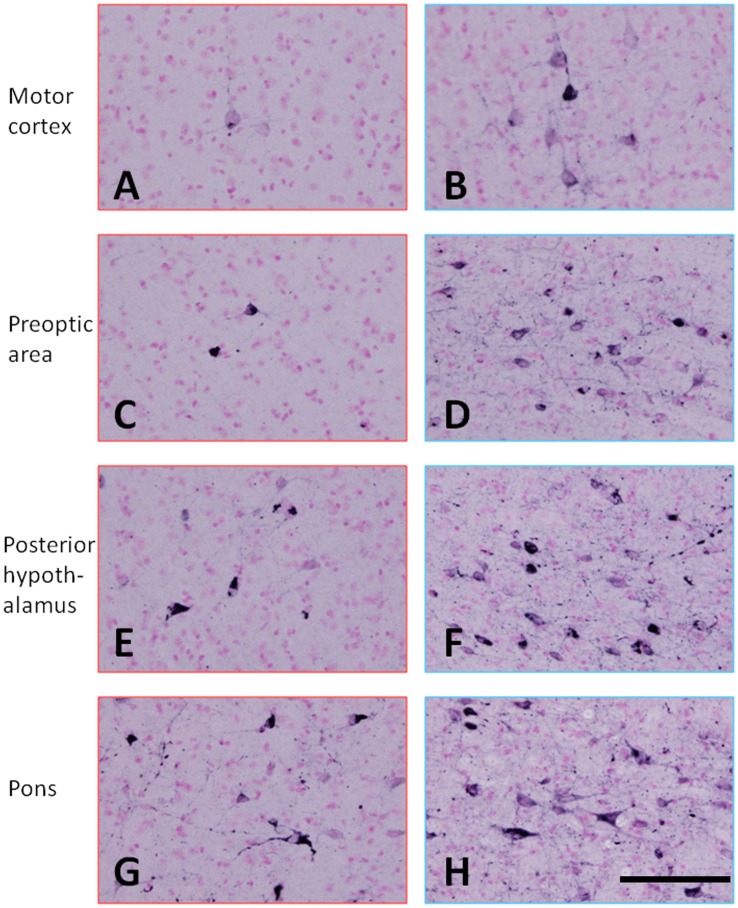
Immunohistochemical staining with a conformational antibody that recognizes aggregated tau. MC-1-positive neurons and cellular processes were seen in the motor cortex (A and B), prepotic area (C and D), posterior hypothalamus (E and F) and pons (G and H). A, C, E, G, mouse with a low AT8/HT7 ratio; and B, D, F, H, mouse with a high AT8/HT7 ratio. The calibration bar in H applies to all photomicrographs (50 µm).

A weak AT8 immunoreaction was observed only in the spinal cord, medulla oblongata and pons of the mice with a low AT8/HT7 ratio (Fig. 5A). On the other hand, AT8-positive cells were seen in the spinal cord, medulla oblongata, cerebellar nuclei, pons, midbrain, hypothalamus and primary motor cortex of mice with a high AT8/HT7 ratio (Fig. 5B). Immunohistochemical staining with a conformational antibody, MC-1, which recognizes aggregated tau showed that MC-1-positive neurons and cellular processes were seen in the motor cortex, hypothalamus and pons (Fig. 6). However, MC-1-positive neurons and cellular processes in these areas of mice with a low AT8/HT7 ratio were significantly fewer than those in mice with a high AT8/HT7 ratio.

## Discussion

Cases of dementia including AD have been increasing in number in recent years, and discovering effective drugs for treating these diseases must be facilitated. Developing a novel drug for the treatment of AD is a time-consuming process and a better approach might be through screening chemical compounds or drugs which are already available. Consequently, we evaluated MB inhibition of abnormal tau accumulation using a tauopathy mouse model, since MB had been shown to be a protein aggregation inhibitor *in vitro* [2] as well as more recently in cells [11].

MB was found to have an inhibitory effect on tau aggregation *in vitro* [1]–[2] and to act as a tau aggregation inhibitor in a cellular model as well as in transgenic mice [6]. However, in the latter case, only an abstract was published on these findings and the details were unavailable. More recently, Congdon et al. reported that MB induced autophagy and attenuated tauopathy in vitro and in vivo [7]. They administrated MB (0.02, 2, and 20 mg/kg) to tau transgenic mice (JNPL3) by oral gavage 5 days a week for 2 weeks and performed immunoblotting for total human tau and sarkosyl-insoluble fractions. There was a significant reduction in the levels of total tau and phosphorylated tau in their MB-treated mice, however, the levels of sarkosyl-insoluble tau were unchanged [7]. In the present study, the immunoblotting data revealed that phosphorylated tau was decreased in the sarkosyl-insoluble fraction after MB administration (Fig. 4). In both soluble and insoluble fractions, the amount of total tau did not differ among the three groups (Fig. 1 and 3), suggesting that the MB administration did not affect the transgenic tau expression but reduced the aggregation of phosphorylated tau. Moreover, immunohistochemical staining demonstrated that MB inhibited the spread of abnormal tau deposition from the brain stem to the cerebral cortex (Fig. 5 and 6). These immunohistochemistry findings supported the results obtained by immunoblotting.

The results of this study may agree with those by Congdon et al. in that the MB treatment reduced phosphorylated tau levels [7]. They also found reduction in the total tau levels in the total but not sarkosyl-insoluble brain fractions. While we did not examine the total brain fractions, we did not see significant changes in the levels of both soluble and insoluble tau between the MB and water treated groups. The reason for such a difference remains unknown, but it might be noteworthy that the duration of MB treatments was quite different between the two studies; we administrated MB 7 days a week for 5 months whereas they did 5 days a week for 2 weeks.

In a study by Medina et al., 0.025% MB was supplied in food to 3×Tg AD mice from 6 to 10 months of age. These mice subsequently showed reduced Aβ levels (soluble Aβ_40_ and Aβ_42_) and were rescued from an early cognitive deficit by an increase in proteasome activity [12]. MB improved learning and memory in these mice and the MB treatment did not affect mitochondrial function or tau pathology. In contrast, Necula et al. demonstrated that MB inhibited Aβ oligomerization by promoting fibril formation in vivo [13].

MB did not affect abnormal tau phosphorylation in a tau transgenic zebrafish model [5]. It also failed to inhibit tau and polyglutamine-protein-dependent toxicity in zebrafish, although polyglutamine aggregation was dramatically reduced. These negative results led to the conclusion that an insufficient dose of MB had been used in vivo [5]. Fertilized eggs of tau transgenic fish were cultured in a buffer containing 10^−5^% MB. However, this transgenic model was not suitable for in vivo analysis of protein aggregation and deposition. Recently, Yamashita et al. reported that MB prevented deposition of TDP-43 in a cell culture model based on ectopic overexpression of TDP-43 [11]. These findings suggested that MB would have an anti-aggregation effect on other neurodegeneration-associated proteins.

Recent reports have indicated that MB affects the brain in many ways: it inhibits butyrylcholinesterase activity [14], inhibits noradrenalin re-uptake [15], reduces cGMP and nitric oxide [16], increases extracellular levels of serotonin (5-HT) in rat brain [12], and modulates the functions of AMPA/kainate and NMDA-type ionotropic glutamate receptors [17]–[18]. The beneficial effect on cognitive function observed in AD patients after MB administration may be in part attributable to its influence on the cholinergic, serotonergic and glutamatergic systems [3]. MB also improves mitochondrial respiration by shuttling electrons to oxygen in the electron transport chain, and corrects perturbations in mitochondrial metabolism induced by mutagens [19]–[20].

Our study suggests that MB has disease-modifying activity in targeting tauopathy involving AD. The administration of MB carried out here was very simple and could easily be applied in screening other compounds for targeting tauopathy. MB could be a leading candidate, and some MB derivatives might be exploited to develop even stronger inhibition of tau aggregation.

MB has already been used for the treatment of methemoglobinemia [21]–[22], septic shock [23]–[24], and Plasmodium infection (malaria) [25]–[26], which may expedite its quick approval as a potential therapeutic agent for tauopathy. While the mechanism involved remains unknown, our data suggest that administration of methylene blue might reduce abnormal tau accumulation. Finally, we did not study pharmacokinetics of MB. Congdon et al. [7] demonstrated the brain concentration of MB increased in parallel with the given doses, but this is an issue that also needs futher, more detailed exploration.
